# Paradoxical epigenetic regulation of XAF1 mediates plasticity towards adaptive resistance evolution in MGMT-methylated glioblastoma

**DOI:** 10.1038/s41598-019-50489-2

**Published:** 2019-10-01

**Authors:** Qiong Wu, Anders E. Berglund, Dapeng Wang, Robert J. MacAulay, James J. Mulé, Arnold B. Etame

**Affiliations:** 1 Departments of Neuro-Oncology, H. Lee Moffitt Cancer Center and Research Institute, 12902 Magnolia Drive, Tampa, FL 33612 USA; 2 Departments of Biostatistics and Bioinformatics, H. Lee Moffitt Cancer Center and Research Institute, 12902 Magnolia Drive, Tampa, FL 33612 USA; 3 Departments of Anatomic Pathology, H. Lee Moffitt Cancer Center and Research Institute, 12902 Magnolia Drive, Tampa, FL 33612 USA; 4 Departments of Immunology, H. Lee Moffitt Cancer Center and Research Institute, 12902 Magnolia Drive, Tampa, FL 33612 USA

**Keywords:** CNS cancer, Tumour-suppressor proteins, Gene silencing, CNS cancer

## Abstract

Epigenetic regulation of *O*^6^-alkylguanine DNA alkyltransferase (MGMT) is surrogate of intrinsic resistance to temozolomide (TMZ). However, mechanisms associated with adaptive resistance evolution of glioblastoma (GBM) relative to MGMT methylation remain unclear. We hereby report a paradoxical yet translational epigenetic regulation of plasticity towards adaptive resistance in GBM. Based on an adaptive resistance model of GBM cells with differential MGMT methylation profiles, MGMT-hypermethylation enhanced genetic and phenotypic plasticity towards adaptive resistance to TMZ while MGMT hypomethylation limited plasticity. The resulting model-associated adaptive resistance gene signature negatively correlated with GBM patient survival. XAF1, a tumor suppressor protein, paradoxically emerged as a mediator of differential plasticities towards adaptive resistance to TMZ through epigenetic regulation. XAF1 promoted resistance both *in-vitro* and *in-vivo*. Furthermore, XAF1 expression negatively correlated with XAF1 promoter methylation status, and negatively correlate with GBM patient survival. Collectively, XAF1 appears to have a pradoxical yet translational role in GBM.

## Introduction

Cancer cells are highly evolved and adapted for survival despite therapeutic and adverse tumor microenvironmental challenges. Sustained cancer therapy can promote resistance through the propagation of highly adaptive tumors or clones contributing to adaptive resistance^1,2^. Glioblastoma multiforme (GBM) is one of the most highly resistant and lethal cancers in adults. Tumor molecular and clonal heterogeneities as well as extensive genome-wide anti-apoptotic molecular aberrations are largely responsible for intrinsic resistance and hence treatment failures in GBM^3,4^. For instance several signaling pathways implicated in GBM oncogenesis and propagation such as EGFR, PDGFR, PI3K, and STAT3 are highly featured in GBM^4^. The Cancer Genome Atlas (TCGA) genetic aberrations in GBM were originally clustered into distict molecular subgroups with differential resistance potential^5,6^, and intratumoral heterogeneity in signaling pathways as well as GBM molecular subtypes is common in GBM even at the single cell level^7^. Disease-control is therefore typically short-lived, with most patients progressing while on maintenance Temozolomide (TMZ) and ultimately succumbing to the disease in approximately 14 months^8^.

The promoter methylation status of the DNA-repair enzyme O6-methylguanine-DNA methyltransferase (MGMT) is a well established clinical indicator of intrinsic resistance to GBM therapy^9,10^. MGMT repairs DNA lesions created by alkylating chemotherapy agents such as TMZ. Hence TMZ is less effective in the presence of increased MGMT activity compared to decreased MGMT activity. MGMT expression is highly regulated through epigenetic mechanisms; hypermethylation of the MGMT promoter results in epigenetic silencing which clinically correlates with response therapy^9,10^. Conversely, hypomethylation of the MGMT promoter is associated with intrinsic resistance to therapy. However, randomized clinical trials designed to target MGMT activity in GBM during TMZ treatment have not been very effective^11,12^. When the MGMT inhibitor O6-benzylguanine was combined with TMZ in a randomized phase II trial, there was no significant difference in efficacy compared to TMZ alone^12^. Furthermore, dose-dense TMZ strategies designed to competively overcome MGMT activity have equally failed to afford superior clinical efficacy when compared to standard dose TMZ in randomized clinical trials^11^. Hence although MGMT promoter methylation status is widely considered as a marker for intrinsic TMZ resistance, the role of epigenetic regulation of adaptive resistance in GBM remains unclear. An enhanced understanding of the evolution of adaptive resistance could provide novel therapeutic avenues to ameliorate treatment failures in GBM.

We have in the past identified and modeled non-epigenetic mechanisms associated with adaptive resistance in GBM^13,14^. We hereby report a paradoxical epigenetic regulation of plasticity towards adaptive resistance in GBM involving XIAP-associated factor 1 (XAF1). XAF1 a previously reported tumor suppressor^15–17^, emerged as a key gene whose epigenetic regulation mediated differential plasticities towards adaptive resistance to TMZ in GBM. XAF1 promotes p53-mediated apoptosis^17^, TNFalpha-mediated apoptosis^16^, and caspase-mediated apoptosis through antagonism of X-linked inhibitor of apoptosis protein (XIAP)^18–22^. Its role as a tumor suppressor protein is further supported by the observations that XAF1 expression is either low or absent in several cancers^23–30^. Most recently it was reported that XAF1 could be epigenetically silenced in high grade glioma and that the methylation status of the XAF1 promoter can serve as a prognostic and/or predictive marker^31^.

Using an adaptive resistance model of GBM cell lines with differential MGMT methylation profiles, MGMT-hypermethylation (MGMT-hyper) enhanced genetic and phenotypic plasticity towards adaptive resistance to TMZ while MGMT hypomethylation (MGMT-hypo) limited plasticity. The resulting model-associated adaptive resistance gene signature negatively correlated with GBM patient survival. XAF1 a tumor suppressor protein, paradoxically emerged as a mediator of differential plasticities towards adaptive resistance to TMZ through epigenetic regulation. XAF1 promoted resistance both *in-vitro* and *in-vivo*. Furthermore, XAF1 expression negatively correlated with XAF1 promoter methylation status, and negatively correlate with GBM patient survival. Collectively, XAF1 appears to have a pradoxical role in GBM that is translational.

## Results

### MGMT-hyper GBM demonstrates enhanced plasticity towards genetic pertubations secondary to sustained therapeutic pressure from TMZ

**Figure 1:**
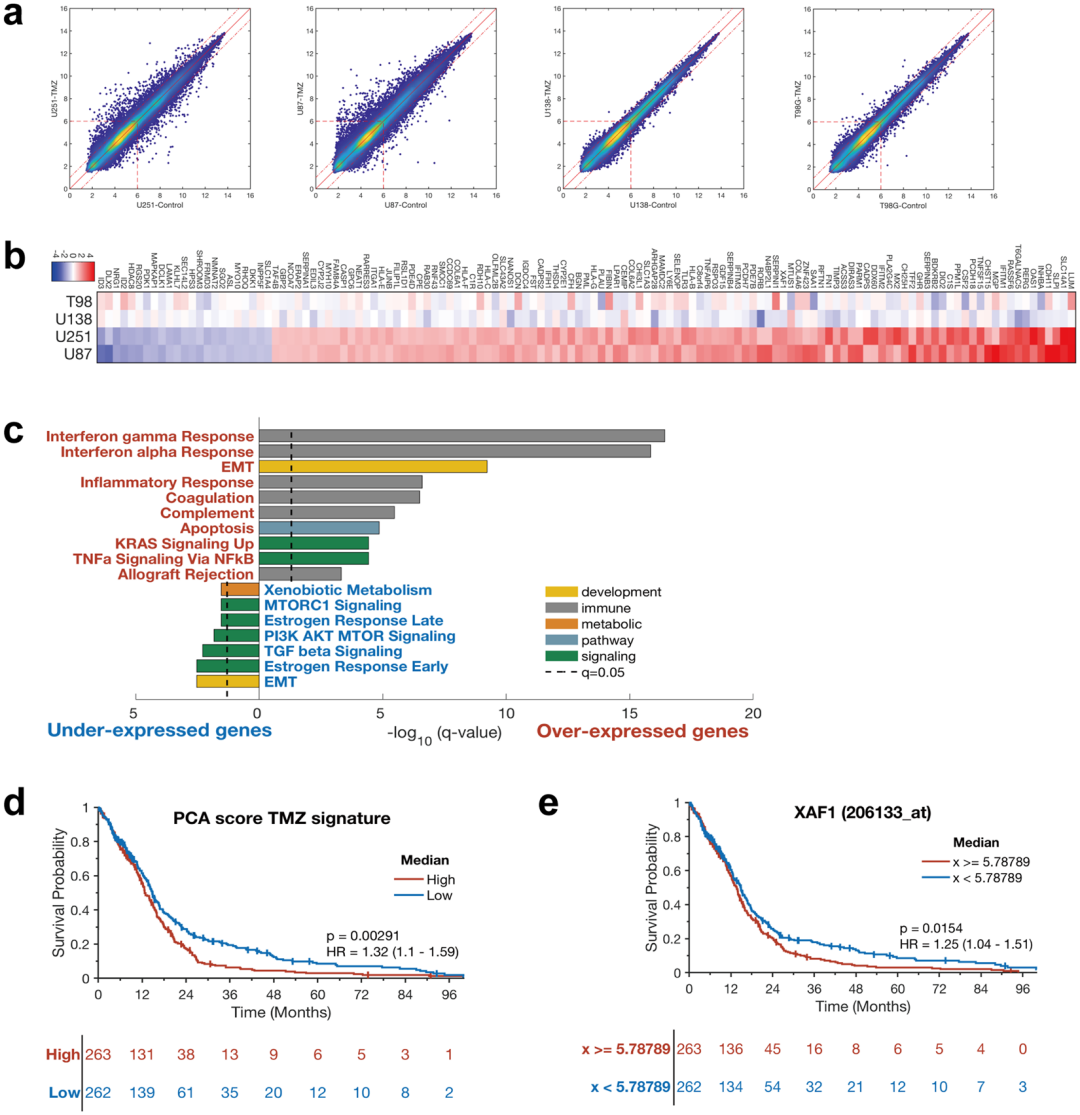
Microarray and gene signature analysis in GBM resistant cells and survival analysis in TCGA GBM. (**a**) The pairwise comparison of the naïve and the resistant cell lines. (**b**) Heatmap using the 129 genes, fold change values are between naïve and treated. (**c**) Gene set enrichment analysis using the Hallmark gene sets for the up-regulated or down-regulated genes and pathways in U251 and U87 cell lines. (**d**) PCA signature from TMZ cell line experiment are related to survival in TCGA GBM data (p = 0.00291). (**e**) TCGA GBM data shows XAF1 expression is significantly related to survival of GBM patients, especially long term (p = 0.0154).

Given that MGMT promoter methylation status in GBM has been postulated as a measure of intrinsic resistance to TMZ^9,10^, we sought to understand the evolution of adaptive resistance relative to intrinsic resistance in MGMT-hyper and MGMT-hypo cells. We therefore examined gene expression changes between adaptively resistant cells and their respective treatment-naïve conterparts using an affymetrix gene expression array (Supplementary Table 1). A PCA plot (Supplementary Fig. 1a) was generated which shows that treatment with TMZ produced only small changes in gene expressions for each cell line. We then focused specifically on genetic changes associated with evolution towards adaptive resistance through comparision of resistant cell lines with treatment-naïve cell lines. Using pairwise comparison, we observed a significantly larger number of genetic perturbations (Fig. 1a) due to TMZ adaptive resistance in human MGMT-hyper GBM cell lines (U251 & U87) compared to MGMT-hypo cell lines (U138 & T98G). Hence, it appears that hypermethylation of MGMT was associated with enhanced genetic plasticity towards adaptive resistance evolution.

In order to further our understanding of genetic alterations associated with adaptive resistance, we generated a heatmap consisting of genes that were either upregulated (red) or downregulated (blue) as GBM cells adapted to evolutionary selection pressure from TMZ (Fig. 1b). The heatmap confirmed that the most upregulation and downregulation of genes occurred with MGMT-hyper GBM cell lines (U251 & U87). The main significantly down-regulated genes adapted to TMZ were associated with the following signaling pathways: EMT, estrogen response, MTORC1, and TGF beta (Fig. 1c). Signaling pathways associated with interferon gamma response, interferon alpha response, EMT, complement, coagulation, inflammatory response, apoptosis, allograft rejection were significantly up-regulated as GBM cell lines adapted to TMZ (Fig. 1c).

We were also interested in determining if differential expression of genes associated with adaptive resistance had any relevance to survival in glioblastoma patients. We generated a PCA score of the top 129 genes associated with TMZ adaptive and examined differential expression of the resulting gene signature in the TCGA GBM expression data set. To validate our signature, based on work by Venet *et al*.^32^ and Berglund *et al*.^33^, we compared it to a random signature generated through random selection of a similar number of genes. Our signature was found to be superior when compared with the random signature in terms of coherence, robutstness, uniqueness, and transferability (Supplementary Fig. 1b). Furthermore, our signature had a lower p log rank value when compared to most random models our signature had (Supplementary Fig. 1c). Our gene signature was statistically significantly associated with overall survival in GBM patients using TCGA data (p = 0.00291, Fig. 1d). Since long-term TMZ treatment can affect the mutational profile of recurrent tumors^34^, we have cross analyzed our gene signature with a signature consisting of genetic alterations from patient tumors treated with TMZ. While there were 58 genes in our signatures that could be possibly affected by long-term TMZ treatment (Supplementary Fig. 1d), there were still 71 genes including XAF1 that were not expected to be affected by long-term TMZ treatment (Supplementary Table 2). When genes associated with TMZ resistance were upregulated, patient survival was markedly shorter compared to genes downregulated. The strong negative correlation between GBM patient survival and genes associated with adaptive resistance supports the validity of our proposed cellular model of adaptive resistance evolution.

In our signature, one of the notably overexpressed genes associated with adaptive resistance to TMZ in MGMT-hyper GBM cell lines was XAF1 (Fig. 1b). This was quite surprising since XAF1 has previously been shown to act as a tumor suppressor protein in several cancers^15–17^. Given the potential paradoxical role of XAF1 expression, we interrogated the TCGA GBM expression dataset to assess what role XAF1 expression had on patient survival. Surprisingly, XAF1 expression actually had a negative correlation with longterm survival in GBM (Fig. 1e). Patients with higher XAF1 expression had a markedly shorter longterm survival compared to patients whose XAF1 expression was lower (p = 0.0154). This suggested that XAF1 may have an aggressive and tumor-promoting role in GBM that was novel and paradoxical to its tumor suppressor role in other cancers.

### MGMT-hyper GBM demonstrates enhanced phenotypic plasticity towards adaptive resistance to TMZ

**Figure 2:**
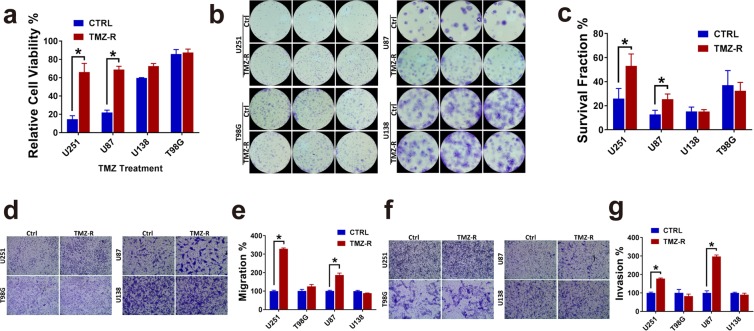
TMZ resistance increased GBM cells viability, proliferation, migration and invasion. (**a**) 1 × 10^3^ U251, U87, U138, T98G control cells or resistance cells were seeded in 96 well plates. Cells were then treated with TMZ (100 μM) for 5 days and cell viability was measured by the XTT Assay. The relative viabilities are shown. With significance for U251, p = 0.009; for U87, p = 0.001. (**b**) The colony forming ability of U251, U87, U138, T98G control cells were compared with resistant cells. (**c**) Clone number calculation in colony formation. With significance for U251, p = 0.017; for U87, p = 0.045. (**d**, **e**) Trans-well migration assay of U251, U87, U138, T98G control versus resistant cells. Cells were induced to move through membranes for 24 hours. Membranes were then fixed, stained and photographed (**d**) or quantitated (**e**). Control cell numbers were normalized as 100%. With significance for U251 p = 1.16E-05; for U87 p = 0.003. (**f**,**g**) Trans-well Invasion assay of U251, U87, U138, T98G control versus resistance cells. Cells were induced to invade through Matrigel-coated membranes for 24 hours. Membranes were then fixed, stained and photographed (**f**) or quantitated (**g**). Control cell numbers were normalized as 100%. With significance for U251 p = 0.0008; for U87 p = 0.0003. All experiments were performed in triplicate and the error bar represent the mean ± SD; n = 3, with significance *p < 0.05 by Student’s t-test.

**Figure 3:**
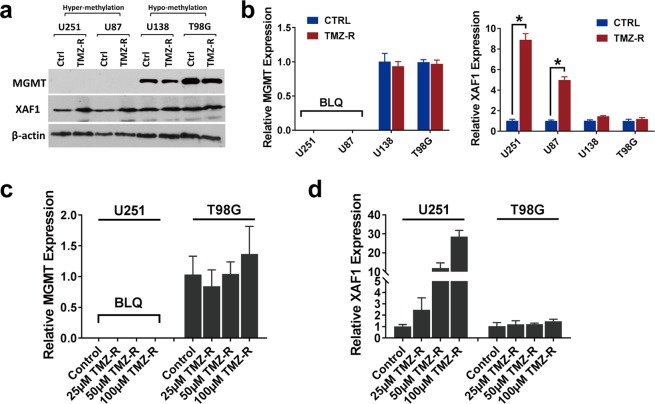
XAF1 plays important role in adaptive TMZ resistance GBM cells independent of MGMT expression. (**a**) Protein expression of MGMT and XAF1 in U251, U87, U138, T98G control and resistant cells. Specific antibodies as indicated. (**b**) MGMT and XAF1 mRNA expression analyzed by real time PCR in U251, U87, U138, T98G control and resistant cells (n = 3, p < 0.05 or no significant difference). BLQ, below limit quantification. (**c**) MGMT mRNA expression level analyzed by real time PCR with U251 & T98G control and TMZ resistance cells, 3 dosages of TMZ resistance were used. (**d**) XAF1 mRNA expression level analyzed by real time PCR with U251 & T98G control and TMZ resistant cells, 3 dosages of TMZ resistance were used. Error bar represent the mean ± SD; n = 3, with significance *p < 0.05 by Student’s t-test.

Based on significant gene expression fold differences between MGMT-hyper cell lines versus MGMT-hypo cell lines (Fig. 1a), we hypothesized that the increased genetic perturbations in MGMT-hyper GBM cell lines were a reflection of plasticity towards adaptive resistance. As a corollary, we speculated that the lesser degree of significant genetic changes observed in the MGMT-hypo cell lines U138 & T98G, was a reflection of limited plasticity potential for these cell lines to develop adaptive resistance to TMZ. In order to test this hypothesis, we examined differential phenotypic manifestations between adaptively resistant cells and their respective treatment-naïve conterparts encompassing both MGMT-hyper and MGMT-hypo GBM cell lines. Resistant and treatment-naïve U251 & U87 cell lines were treated with 100 µM TMZ and analyzed by XTT, there was a significant difference (p < 0.05) in viability between the adaptively resistant and treatment-naïve cells suggesting enhanced plasticity towards adaptive resistance in MGMT-hyper cell lines (Fig. 2a). However, when resistant and treatment-naïve cells U138 & T98G were treated with 100 µM TMZ, there was no significant difference in viability suggesting limited plasticity towards adaptive resistance (Fig. 2a). Next we performed colony formation assay on all cell lines above (Fig. 2b,c). There was a significant difference (p < 0.05) in colony formation between adaptively resistant cells and treatment-naïve cells in U251 and U87. No significant differential response in colony formation was seen in U138 and T98G cells. Transwell migration assays were performed comparing the migratory potential of adaptively resistant and treatment-naïve cells (Fig. 2d,e). There was a significant difference (p < 0.05) in migration between adaptively resistant cells and treatment-naïve cells in U251 and U87. MGMT-hyper GBM cell lines in essence became more migratory as they evolved towards adaptive resistance. U138 and T98G did not exhibit differences in migration between resistant cells and treatment-naïve cells, supporting the notion that MGMT-hypo cell lines have limited or no plasticity to promote further evolution to a highly migratory phenotype. Furthermore, we also compared the invasive potential of adaptively resistant cells and treatment-naïve cells (Fig. 3f,g). Similar to the results with migration, there was a significant difference (p < 0.05) in invasion between adaptively resistant cells and treatment-naïve cells in U87 & U251. MGMT-hyper GBM cell lines became more invasive as they acquired resistance to TMZ.

### XAF1 expression is influenced by differential plasticity of MGMT-hyper and MGMT-hypo GBM during evolution towards adaptive resistance

XAF1 was one of the highly overexpressed genes associated with adaptive resistance to TMZ in MGMT-hyper GBM cell lines (Fig. 1b). Furthermore, increased XAF1 expression in resistant cell lines coupled with the inverse correlation of XAF1 expression with GBM survival (Fig. 1e), suggested that XAF1 could contribute to an aggressive phenotype in GBM which is paradoxical to its tumor suppressor role. In order to further confirm the expression array findings, we examined and compared MGMT and XAF1 protein expression before and after sustained TMZ treatment in our GBM cells by Western blot (Fig. 3a). MGMT protein levels did not significantly change during adaptive resistance evolution of MGMT-hyper GBM and MGMT-hypo cell lines. In contrast, XAF1 protein expression increased during evolution of MGMT-methylated GBM cell lines towards adaptive resistance but remained unchanged in MGMT-hypo cells. We also examined gene expression by real time PCR. MGMT gene expression was not affected as cells adapted to resistance (Fig. 3b). MGMT-hyper GBM cell lines demonstrated plasticity in XAF1 gene expression resulting in upregulation during evolution of adaptive resistance. On the contrary, MGMT-hypo cell lines did not demonstrate any significant plasticity in XAF1 gene expression during adaptive resistance (Fig. 3b). We then proceeded to assess various doses of TMZ (25 µM–100 µM) to ascertain if there was a dose-dependent effect on evolutionary plasticity of MGMT and XAF1 gene expressions (Fig. 3c,d). MGMT-hyper GBM cell lines (U251) demonstrated dose-dependent plasticity to XAF1 expression (Fig. 3d) but not to MGMT expression (Fig. 3c). However, we did not observe any significant dose-dependent modulation of either MGMT or XAF1 expressions in MGMT-hypo cell lines (T98G) (Fig. 3c,d). Although, clonal plasticity to adaptive resistance was dependent on MGMT promoter methylation status, it is interesting to note that MGMT protein and gene expression did not significantly change as a consequence of adaptive resistance. Furthermore, the modulation of XAF1 expression in MGMT-hyper GBM during evolution towards adaptive resistance, in conjunction with the observation that XAF1 expression negatively correlates with longterm survival in GBM (Fig. 1e), support our hypothesis that XAF1 might have an aggressive role in GBM that is paradoxical to its role as a tumor suppressor in other cancers.

### Differential modulation of epigenetic regulation of XAF1 correlates with plasticity of MGMT-hyper and MGMT-hypo GBM towards adaptive resistance

**Figure 4:**
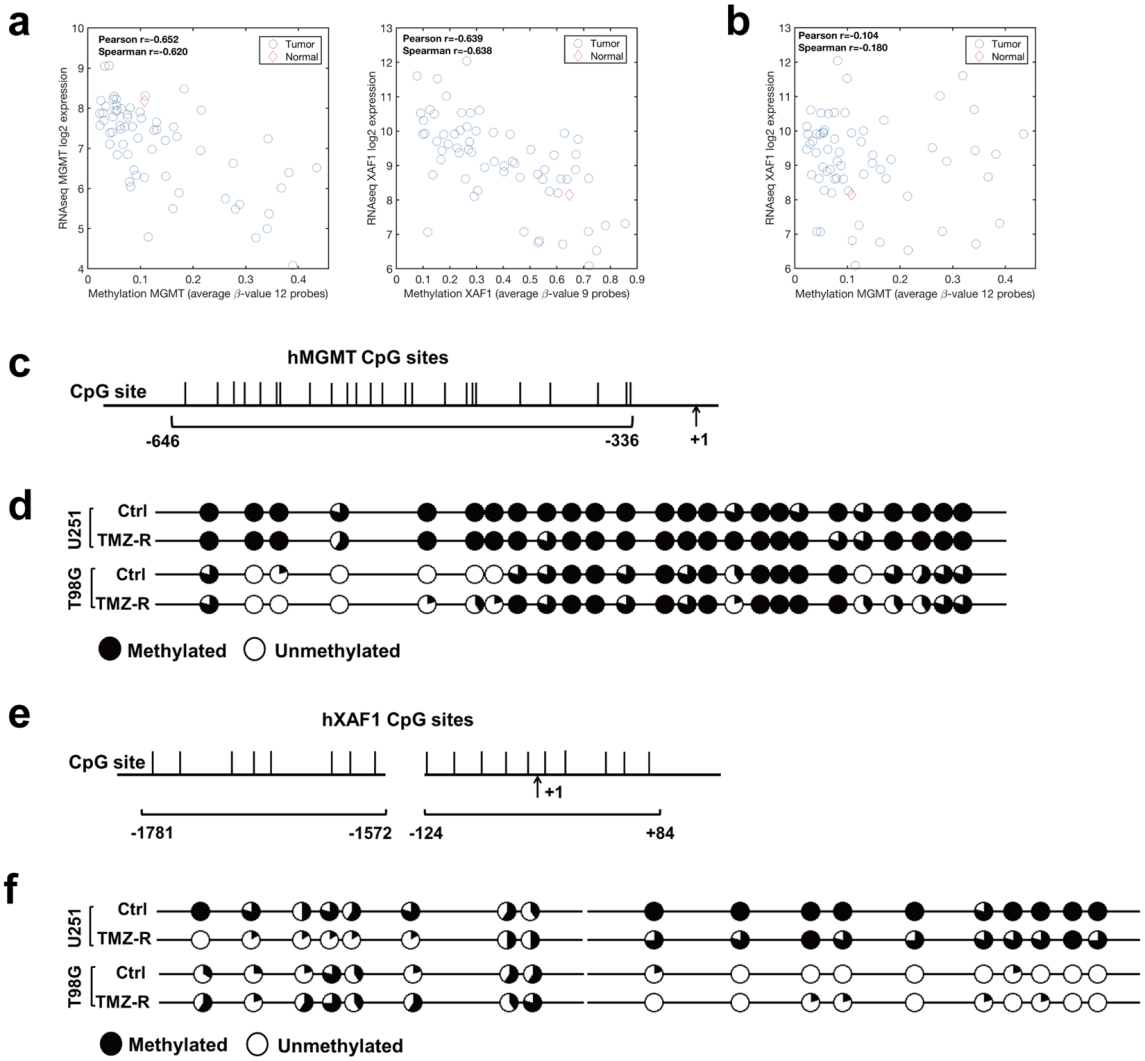
XAF1 but not MGMT promoter was demethylated in adaptive TMZ resistance GBM cells. (**a**) MGMT and XAF1 methylation pattern and correlation with gene expression level. (**b**) Methylation level of MGMT is not correlated to the expression of XAF1. (**c**) Schematic representation of hMGMT promoter shows the location of 25 CpG-dinucleotides sites from −336 to −646 relative to the TSS. TSS, transcription start site. (**d**) Bisulfite sequencing was performed of hMGMT promoter in U251, T98G control and resistant cells. (**e**) Schematic representation of hXAF1 promoter shows the location of 18 CpG-dinucleotides sites from + 84 to −124 and −1572 to −1781 relative to the TSS. (**f**) Bisulfite sequencing result of hXAF1 promoter in U251, T98G control and resistant cells. Each circle represents the average methylation of 5 clones. A hollow circle means no methylation, whereas a filled circle means 100% methylated.

Based on earlier observations related to XAF1, we were interested in determining if modulation of its epigenetic regulation accounted for the differential plasticities in XAF1 expression during adaptive resistance in GBM. We hypothesized that the modulation of promoter methylation of XAF1 likely occured as a consequence of adaptive resistance to TMZ. First, we analyzed the TCGA methylation and RNAseq gene expression data for both MGMT (Supplementary Fig. 2a) and XAF1(Supplementary Fig. 2b). Using the average β-value for 12 probes that span the promoter region of MGMT, we observed a strong correlation between MGMT promoter methylation pattern and MGMT gene expression (Fig. 4a). We also observed a strong correlation between XAF1 promoter methylation pattern and XAF1 gene expression with correlation coefficient similar to that of MGMT, suggesting that both MGMT and XAF1 are highly regulated through methylation status of their respective promoters (Fig. 4a). No significant correlation between MGMT promoter methylation and XAF1 expression was observed (Fig. 4b). It does appear that XAF1 expression is highly regulated through methylation of its promoter, and also XAF1 expression is not directly governed by MGMT methylation status. We then proceeded to examine the methylation status of XAF1 and MGMT promoters during adaptive resistance in MGMT-hyper and MGMT-hypo GBM cell lines. For MGMT promoter, we focused 25 CpG-dinucleotides sites from −336 to −646 relative to the transcription start site (TSS) (Fig. 4c). Based on bisulfite sequencing analysis, MGMT promoter demonstrated no significant change in methylation status when either MGMT-hyper (U251) or MGMT-hypo (T98) GBM cell lines transitioned from the treatment naïve (Ctrl) to adaptively resistant (TMZ-R) status (Fig. 4d). For XAF1 promoter methylation status analysis, we examined 18 CpG-dinucleotides sites from 2 separate CpG island regions, +84 to −124 and −1572 to −1781 relative to the TSS (Fig. 4e). Based on bisulfite sequencing analysis, XAF1 promoter methylation status transitioned from a hypermethylated to a hypomethylated status when MGMT-hyper (U251) GBM cell lines transitioned from the treatment naïve (Ctrl) to adaptively resistant (TMZ-R) status, especially in −1572 to −1781 region (Fig. 4d). XAF1 promoter methylation status of MGMT-hypo (T98G) GBM cell lines was not significantly affected by the transition from treatment naïve (Ctrl) to adaptively resistant (TMZ-R) (Fig. 4f). Taken together, the epigenetic observations with respect to promoter methylation status strongly suggests differential plasticities to XAF1 upregulation as a response to adaptive resistance of MGMT-hyper and MGMT-hypo GBM cell lines.

### Genetic modulation of XAF1 contributes to *in-vitro* adaptive resistance evolution in MGMT-hyper but not MGMT-hypo GBMs

**Figure 5:**
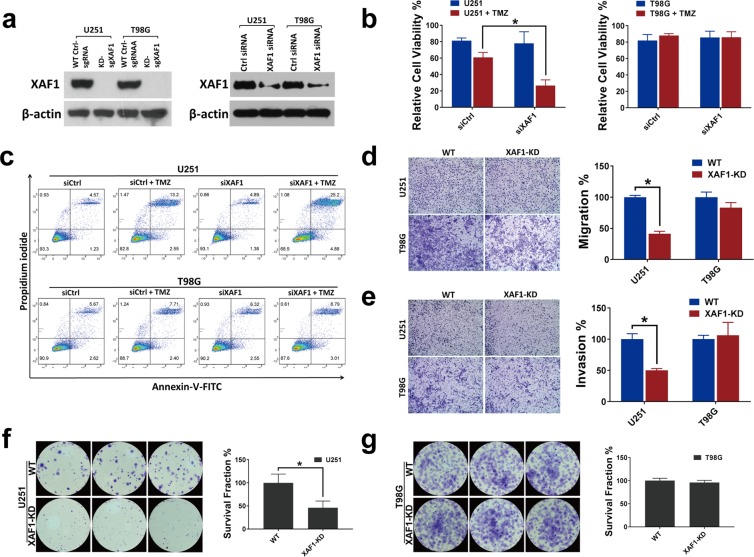
Loss function of XAF1 leads to biological behavior changes in the presence of TMZ. (**a**) Western blot analysis using whole cell lysate derived from wild type control, XAF1 CRISPR/Cas9 knockdown (XAF1-KD), siRNA control and siXAF1 knockdown in U251 and T98G cells. (**b**) 1 × 10^3^ U251, T98G control and siRNA knockdown cells were seeded in 96 well plates. Cells were then treated with TMZ (50 μM) for 5 days and cell viability was measured by the XTT Assay. The relative viability is shown; n = 3, with significance, p = 0.02. (**c**) U251, T98G cells were seeded in 12 well plates overnight. Cells were then knocked down by control siRNA (siCtrl) and XAF1 siRNA (siXAF1), 24 hours later treated with 50 μM of TMZ for 5 more days. Apoptosis was measured and quantified by Annexin V/PI staining through flow cytometry. (**d**,**e**) Trans-well migration and invasion assay of U251, T98G wild type control and XAF1 silenced (XAF1-KD) cells. Cells were induced to move through uncoated/coated membranes. Membranes were then fixed, stained, photographed and quantitated. n = 3; with significance, for migration p = 0.002 and for invasion p = 0.004. (**f**,**g**) The colony forming ability of U251, T98G wild type control was compared with XAF1 silenced (XAF1-KD) cells in presence of 50 μM TMZ. n = 3, with significance, for U251, p = 0.007. All experiments were performed in triplicate and error bar represent the mean ± SD; n = 3, with significance *p < 0.05 by Student’s t-test.

Since there was differential epigenetic modulation of XAF1 expression during evolution of adaptive resistance, we sought to determine the role of XAF1 on the differential adaptive phenotypes of the respective cells. We had noted that MGMT-hyper and MGMT-hypo GBM cells demonstrated differential plasticities to adaptive resistance whereby MGMT-hyper cells were most susceptible to adaptive resistance. For loss of function model, we genetically silenced XAF1 in U251 and T98G cells as stable knockdown (XAF1-KD) by CRISPR/Cas9 technique and demonstrated XAF1 expression with Western blot (Fig. 5a). Here, CRISPR system manufactured GBM cell lines were used as stable knockdowns (loss of function model) but not clonal knockouts. CRISPR/Cas9 editing indels were further confirmed by Sanger Sequencing (Supplementary Fig. 3a,b). With siRNA knockdown, we noted a significant decrease (p < 0.05) in cell viability when XAF1 was silenced in U251 cell line treared with 50 µM TMZ, whereas XAF1 silencing had no significant impact in T98G cells (Fig. 5b). Next, we examined XAF1 silenced U251 and T98G cells with or without TMZ (50 µM) treatment by Annexin-V/PI assay. There was an increase on apoptotic fraction in U251 siXAF1 silenced cells when treated with TMZ, While XAF1 silencing had no effect on apoptotic fractions in T98G cells (Fig. 5c). We then performed Transwell migration and invasion assays in U251 and T98G XAF1 silenced and wildtype control cells that were pre-treated with 50 µM TMZ. Migration and invasion were significantly impaired in U251 XAF1 silenced (XAF1-KD) cells (p < 0.05), while XAF1 silencing had no significant effect on migration and invasion in T98G cells (Fig. 5d,e). Lastly, we examined the ability of our XAF1 manipulated cells to form colonies when treated with TMZ. Colony formation was significantly impaired when XAF1 was silenced in U251 cells treated with TMZ (Fig. 5f). XAF1 silencing had no significant effect on colony formation in T98G cells when treated with TMZ (Fig. 5g).

Since our data suggested that silencing of XAF1 limited the ability of MGMT-hyper GBM cell lines to become adaptively resistant to TMZ, we also wondered if silencing of XAF1 in the cells that were already adaptively resistant could reverse resistance to TMZ. We silenced XAF1 by CRISPR/Cas9 technique as stable knockdowns of both U251 TMZ-R and T98G TMZ-R cells that were already adaptively resistant to TMZ (Supplementary Fig. 4a). Similar to our results above with treatment naïve GBM cells, we noted a significant decrease (p < 0.05) in cell viability when XAF1 was silenced in U251 TMZ-R cells, whereas XAF1 silencing had no significant impact on the viability of T98G TMZ-R cells treated with TMZ (Supplementary Fig. 4b). On flow cytometry analysis, there was a significant increase (p < 0.05) in apoptotic fraction of U251 TMZ-R cells treated with TMZ when XAF1 was silenced (Supplementary Fig. 4c). Similar to prior observations, XAF1 silencing had no significant impact on the apoptotic fraction of T98G TMZ-R cells (Supplementary Fig. 4c). When we assessed migration and invasion through Transwell assay, there was a significant decrease (p < 0.05) in both migration and invasion when XAF1 was silenced in U251 TMZ-R cells were treated with TMZ, whereas XAF1 silencing had no significant effect in T98G TMZ-R cells (Supplementary Fig. 4d,e). We also assessed colony formation in XAF1 modulation of the adaptively resistant GBM cells during TMZ treatment. Colony formation was significantly impaired (p < 0.05) when XAF1 was silenced in U251 TMZ-R cells (Supplementary Fig. 4f), whereas no significant effect in T98G TMZ-R cells (Supplementary Fig. 4g).

### Genetic silencing of XAF1 contributes *in-vivo* to TMZ sensitivity in MGMT-hyper but not MGMT-hypo GBM

**Figure 6:**
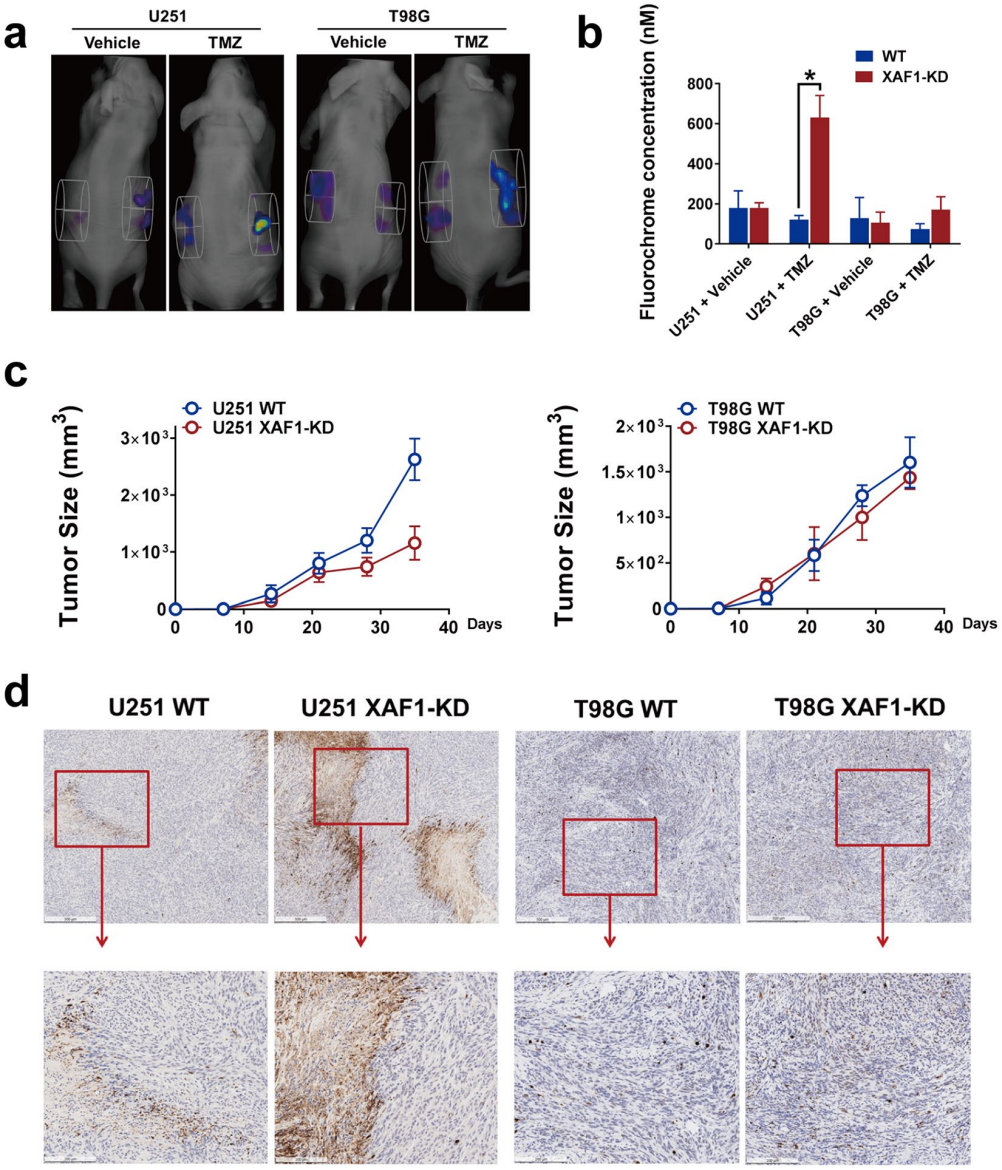
XAF1 expression correlated with TMZ induced apoptosis *in vivo*. Mouse GBM xenografts were set up by injecting 4 × 10^6^ GBM wild type control (WT) or XAF1 silenced (XAF1-KD) cells on the left and right flank, respectively, of the same mice. Three weeks later, mice were treated with either 50 mg/kg of TMZ orally or vehicle control daily for 7 days. Mice were then injected with 50 μ L NIR-FLIVO747 via the lateral tail vein, 4 hours later, mice were imaged using the PerkinElmer FMT2500 to compare tumor cell apoptosis on both sides. (**a**) For each mouse, left flank: wild type cells, right flank: XAF1 silenced (XAF1-KD) cells. (**b**) Average fluorochrome concentration in the tumor area on both sides. Error bar represent the mean ± SD; n = 5, with significance *p < 0.05 by Student’s t-test. (**c**) Tumor growth curves of U251/T98G control and XAF1 silenced (XAF1-KD) tumors. (**d**) Mice were sacrificed and tumor tissue on both sides was isolated, cut and fixed in 10% neutral formalin. Cleaved Caspase-3 immunohistochemistry was performed as described in the Material and Methods section. From left to right: U251 wild type (U251 WT), U251 XAF1 silenced (XAF1-KD), T98G wild type (T98G WT) and T98G XAF1 silenced (XAF1-KD) tumors. 5 mice were included in this study and similar results were observed in each animal.

In an effort to ascertain if our *in vitro* above findings on XAF1 were applicable in xenografts, we assessed if XAF1 silencing had any impact on TMZ sensitivities of the MGMT-hyper and MGMT-hypo cell lines in subcutaneous GBM xenografts models. For all animals, we implanted the wild-type GBM cells on the left flank while the XAF1 silenced GBM cells was implanted on the right. Animals were treated daily with either TMZ or vehicle for 7 days after 3 weeks of implantation and then subjected to non-invasive *in vivo* apoptosis imaging for detection of caspase-mediated apoptosis fluorescence. Treatment with vehicle had no significant apoptotic effects on either WT (left flank) or XAF1 silenced (XAF1-KD, right flank) GBM xenografts (Fig. 6a,b). XAF1 silencing resulted to significantly enhanced (p < 0.05) caspase activation and apoptosis in U251 xenografts but not in T98G xenografts following TMZ treatment (Fig. 6a,b). Most important, the XAF1 silenced U251 tumors showed significantly slower growth kinectics compared to U251 wild type tumors (Fig. 6c). On the other hand, T98G tumors didn’t show any significant differences in growth kinetics between wild type control and XAF1 silenced group (Fig. 6c).

We further corroborated our *in vivo* apoptosis imaging findings with immunohistochemistry staining of tumors for cleaved caspase 3. In response to TMZ treatment, there was no significant activation of caspase 3 in neither WT control or XAF1 silenced (XAF1-KD) T98G GBM xenografts (Fig. 6d). While there was caspase 3 activation in both WT and XAF1 silenced (XAF1-KD) U251 GBM xenografts, caspase 3 was more markedly (p < 0.05) activated in XAF1 silenced group compared to WT control group (Fig. 6d). The xenograft results implicate XAF1 as a facilitator of adaptive resistance based on MGMT promoter methylation whereby MGMT-hyper cells demonstrate more plasticity to XAF1 mediated adaptive resistance to TMZ.

## Discussion

Clonal evolution towards a more resistant phenotype following therapeutic intervention is a hallmark of cancers^1,2^, notably GBM. TMZ is the established bona fide first line chemotherapy agent for patients with GBM. However, even when TMZ is combined with radiotherapy following maximum-safe surgical resection, the median survival still remains dismal at 14 months secondary to adaptive resistance to therapy^8^. MGMT promoter hypermethylation is associated with a favorable response to TMZ therapy whereas MGMT promoter hypomethylation is associated with a lesser durable response to TMZ. However, data from randomized clinical trials have not demonstrated any significant synergistic durable response benefits associated with targeting of MGMT activity in GBM during TMZ treatment^11,12^. Hence, while epigenetic regulation of MGMT might serve as a biomarker for resistance, MGMT protein activity does not appear to be a critical driver of adaptive resistance. The present study was therefore undertaken to examine adaptive resistance evolution to TMZ in MGMT-hyper and MGMT-hypo GBM cell lines in an attempt to clarify potential drivers of adaptive resistance in GBM.

In the current study, we employed a model of GBM adaptive resistance evolution to TMZ, and we observed that MGMT-hyper GBM cells were more permissive to adaptive resistance compared to MGMT-hypo GBM cells. A genetic signature of adaptive resistance from our model showed a very strong inverse correlation with long-term survival in the GBM patient TCGA dataset suggesting our model of adapative resistance evolution was clinically relevant. This analysis was also consistent with previous reports that high levels of XAF1 is associated with poor patient survival^31^. Moreover, we report a novel paradoxical role of XAF1 in GBM adaptive resistance whereby, XAF1 expression through differential epigenetic regulation promotes both genetic and phenotypic resistance in MGMT-hyper GBM cell lines during TMZ treatment. In fact, although previously described as a tumor suppressor protein in several cancers, we observed a strong inverse correlation between XAF1 expression and longterm survival in the GBM patient TCGA dataset. Patients with relatively increased XAF1 expressions had a shorter survival. Our findings on XAF1 and permissivity of MGMT-hyper GBM cell lines towards adaptive resistance in GBM are novel with several interesting implications.

First our observation that MGMT promoter hypermethylation is associated with enhanced plasticity or permissivity towards adaptive resistance appears counterintuitive since hypermethylation is routinely clinically viewed as a favorable biomarker in GBM. Hypermethylated GBM cell lines are less intrinsically resistant to TMZ than MGMT-hypomethylated cells based on IC-50 values. However, upon sustained TMZ treatment, both genetic and phenotypic plasticities towards adaptive resistance were observed in MGMT promoter hypermethylated GBM cell lines whereas MGMT-hypomethylated cells demonstrated very limited plasticity to further adaptive resistance. We suggest that the baseline relatively low intrinsic resistance of MGMT-hyper cells makes them more prone to evolution towards a more aggressive phenotype during TMZ treatment. On the other hand, since MGMT-hypo cells have a higher intrinsic resistance to TMZ, sustained TMZ exposure does not confer any significant adaptive resistance. Clinically, it would therefore appear that MGMT-hyper GBMs are most amenable to TMZ treatment strategies that modulate adaptive resistance. Similarly, strategies that target genetic drivers with of adaptive resistance to TMZ could be most effective in terms of synergy with TMZ in MGMT-hypermethylated GBMs.

Second, although MGMT promoter methylation status and hence expression have been postulated as a clinical indication of intrinsic resistance to TMZ, we did not observe any significant changes in MGMT expression during evolution towards adaptive resistance irrespective of the MGMT promoter methylation status. Hence, direct targeting of MGMT might not prevent adaptive resistance evolution to TMZ. In addition, the above findings could explain why inhibition of MGMT activity did not provide synergy towards durable TMZ response in clinical trials^11,12^.

The most interesting observations related to the paradoxical function of XAF1, a previously reported tumor suppressor gene whose expression could induce apoptosis in tumor cells^15–17^. The tumor suppressor role of XAF1 is well established in several gastrointestinal cancers, melanoma, and urogenital cancers, where its expression levels are documented to be extremely low^23–29^. It appears XAF1 through unclear mechanisms promotes adaptive resistance and aggressive tumor phenotype in GBM when challenged with TMZ. Further, genetic silencing of XAF1 expression actually sensitized MGMT-hyper GBM cells to TMZ. It is also apparent that beyond adaptive resistance to sustained TMZ challenge, XAF1 could have a distinct role in GBM biology in general. When we examined XAF1 expression in newly diagnosed GBM patients through the TCGA data, there was an inverse correlation between XAF1 expression and long-term survival. Patients with relatively higher XAF1 expression had a markedly lower long-term survival suggesting that XAF1 might not actually play in tumor suppressive or an apoptosis promoting role in GBM as is the case in other cancers. Our findings are further supported by previous studies that examined the relationship between epigenetic silencing of XAF1 and outcomes in patients with high grade gliomas^31^. It was noted that promoter hypermethylation of XAF1 was associated with a better prognosis and clinical outcome in high grade gliomas compared to XAF1 promoter hypomethylation^31^. Hence silencing of XAF1 expression was associated with a favorable outcome. Furthermore, there was a strong correlation between XAF1 promoter methylation and IDH mutations^31^. Further studies are certainly warranted to fully understand how mechanistically XAF1 promotes GBM tumorigenesis.

Lastly, our model of adaptive resistance was mainly a cellular model that has limitations related to *in-vitro* artifacts. GBM cell lines were selected that represented MGMT-hypo and MGMT-hyper phenotypes and challenged with TMZ in a sustained fashion. Although the cell lines might not entirely recapitulate patient-derived GBMs, the gene signature for adaptive resistance that emerged from our cellular model correlated as expected with TCGA data. This signature had an impact on patient survival suggesting potential clinical relevance and the value of such *in-vitro*-models. Clinically, It has been reported that long term treatment of TMZ will cause genomic alterations, at least in part, driving the growth of recurrences that are distinct from genomic alterations in the initial tumor^34^. Those *de novo* mutations could drive the evolution of TMZ-resistant GBM cells to more aggressive potential. However, in our TMZ-resistant cell model, although 58 of the 129 genes in our signatures could be possibly affected by TMZ treatment (Supplementary Fig. 1d), there are still 71 genes including XAF1 that would not be affected by TMZ treatment (Supplementary Table 2). Lastly, we employed a flank model with *in-vivo* apoptosis detection capabilities as the most practical approach to established an *in-vivo* proof-of-principle with respect to the role XAF1 to TMZ responsiveness.

In summary, our studies have shed some light with respect to the plasticity of GBM towards adaptive resistance evoluation to TMZ relative to MGMT promoter methylation status. In particular, a novel and translational role for XAF1 in GBM has been uncovered. Further studies of XAF1 and other potential candidates associated with our adaptive resistance evolution signature will undoubtedly provide new avenues for therapeutic synergies with TMZ especially for MGMT-hypermethylated GBM.

## Methods and Materials

### Glioblastoma multiforme cell lines TMZ resistance microarray

Total RNA was extracted using RNeasy mini-prep kit (Qiagen) and followed by DNase digestion using RNase-Free DNase Set (Qiagen). Extracted RNA was labeled and hybridized onto the GeneChip PrimeView Human gene expression array cartriade (Affymetrix, United States).

### Mice

6–8 weeks female NCRNU athymic mice were ordered from Taconic Biosciences. All animals were housed in the American Association for Laboratory Animal Care–accredited Animal Resource Center at Moffitt Cancer Center. All animal procedures and Experiments were carried out under protocols approved by the Institutional Animal Care and Use Committee of the University of South Florida and Moffitt Cancer Center. All animal studies were performed in accordance with relevant guidelines and regulations of University of South Florida and Moffitt Cancer Center.

### Cell culture

U251, U87, U138 and T98G human GBM cells (ATCC) were cultured in DMEM (Life Technologies) supplemented with 10% fetal bovine serum (Sigma-Aldrich), 100 units/ml penicillin-100 μg/ml streptomycin (Life Technologies). The cultures were maintained at 37 °C in a humidified atmosphere containing 5% CO_2_. TMZ-resistant GBM cells were generated as previously described^13^.

### Real-time PCR

Total RNA was extracted using RNeasy mini-prep kit (Qiagen). RNA was quantified with Nanodrop 2000 (Thermo Scientific). cDNA was synthesized using 1 μg total RNA with the iScript cDNA Synthesis Kit (Bio-Rad). Real-time PCR was performed using the Bio-Rad CFX96 Touch Real-Time PCR Detection system. *MGMT* forward primer: GGGTCTGCACGAAATAAAGC, reverse primer: GAAATAGGCATTCAGCCAGG; *XAF1* forward primer: ATGGAAGGAGACTTCTCGGT; reverse primer: TTGCTGAGCTGCATGTCCAG. *GAPDH* was used as the internal control, *GAPDH* forward primer: ACCACAGTCCATGCCATCAC, reverse primer: TCCACCACCCTGTTGCTGT. The PCR program was as follows: 95 °C 10 minutes, 1 cycle; 95 °C 15 seconds −>60 °C 30 seconds −>72 °C 30 seconds, 40 cycles; 72 °C 10 minutes, 1 cycle.

### XTT viability assay

Viability of cells was measured using an XTT Cell Viability Assay Kit (Cell Signaling). 50 μl of XTT detection solution was added to each well of a 96-well plate (containing 200 μl/well of culture medium) following treatment/transfection of cells, and incubated at 37 °C for 2 hours. The absorbance was then measured at 450 nm using a microplate reader (Molecular Device, USA). The relative survival was calculated by dividing the absorbance of experimental group with that of the control group.

### Clonogenic assay

1000–2000 cells were seeded in 6-well plates. The cells were then cultured until day 14. The culture medium was removed and fixed with ethanol. Cell clones were stained with crystal violet (EMD, USA) for 30 minutes. The plates were gently washed with water and dried at room temperature overnight. Cell clones were then counted under a light microscope. A cell clone was defined to include at least 50 cells. The plating efficiency (PE) was calculated by: PE = 100 × number of clones counted/number of cells plated. The survival fraction (SF) was calculated by: SF = 100 × PE of treated group/PE of control group.

### Genomic DNA extraction and bisulfite DNA sequencing analysis

gDNA was extracted from either GBM cells or GBM TMZ-resistance cells using the Quick-DNA Miniprep kit (Zymo). gDNA was bisulfite converted using EpiTect Fast DNA Bisulfite Kit (Qiagen) according to the manufacturer’s protocol. The bisulfite-converted DNA was subjected to PCR amplification using specific primers to MGMT and XAF1 promoter. For MGMT, we used the following primers to amplify a 310 bps region of the promoter CpG island, forward: TTGAGTTAGGTTTTGGTAGTGTTTAG, reverse: CCTTTTCCTATCACAAAAAT AATCC; we used the following primers to amplify 2 regions from XAF1 promoter CpG island. For 275 bps region forward: GGGAGGGTTTAGTTTTAGGAATTAA, reverse: ACTCCCTTCAAAAACAACCCTATAT; for 209 bps region, forward: TTATTGTAGGTTTTAT TTTGGGTTTA, reverse: TCATACCTATAATCCCAACCCTTTA. PCR products were gel purified and cloned using pGEM-T Easy Vector System (Promega), and positive clones DNA were extracted by Qiaprep Spin Miniprep Kit (Qiagen) and sent for sequencing. Sequencing analysis was done using Quantification tool for Methylation Analysis (QUMA: http://quma.cdb.riken.jp).

### Trans-well migration and invasion assay

The trans-well migration and invasion assay were carried out using 24 mm Transwell^®^ Cell Migration and Invasion assay kits (Corning). 4 × 10^4^ cells (normal cultured or pre-treated) were seeded and incubated in trans-well, 24–48 hours for migration and invasion. Migrating and transversed cells were fixed with ethanol and then stained with crystal violet. The number of migrated and invaded cells were quantified by counting under a light microscope. All experiments were done in triplicate.

### Flow cytometry for apoptosis analysis

Annexin V/PI assay was used to test cell apoptosis through flow cytometry. GBM cells were harvested and washed by PBS. Then cells were stained with Annexin V-FITC (BD Biosciences) in staining buffer and stained with PI (Sigma-Aldrich) in PBS. The stained cells were immediately tested using a BD LSRII flow cytometer and data was analyzed by FlowJo software (Tree Star, USA).

### GBM xenograft model

4 × 10^6^ U251 wild type control or XAF1 silenced (XAF1-KD) cells were mixed with Matrigel (Corning, USA) and injected at both flanks of NCRNU athymic mice. All the mice were treated with TMZ after three weeks since injection (50 mg/kg per day, orally).

### Apoptosis detection through live animal imaging

*In vivo* apoptosis of the flank tumor set up above was monitored using NIR-FLIVO 747 *in vivo* Apoptosis Kit (ImmunoChemistry Technologies, USA). NIR-FLIVO were injected through the lateral tail vein of the mice and allowed to circulate in the mice for optimal image acquisition. Four hours later, animals were anesthetized and imaged by FMT-2500LX (PekinElimer, USA).

### Immunohistochemistry

Tumor samples from mice flank model were fixed with 10% NFB (neutral-formalin buffer) for 48–72 hours, dehydrated, paraffin-embedded and sectioned. Once deparaffinized and treated with H_2_O_2_, sections were incubated with cleaved Caspase-3 antibody (1:100 dilutions) at 4 °C overnight. The sections were then incubated with secondary antibody (1:200 dilutions) and followed by ABC-peroxidase incubation. The sections were washed with Tris-buffer, incubated with DAB (3, 30 diaminobenzidine), rinsed and stained with hematoxylin.

### Bioinformatics

Human GBM TMZ treated cell lines. The Affymetrix U133 plus 2.0 CEL files were normalised using IRON^35^ and log2 transformed before analysis. The following criteria were used for selecting the most important genes: The fold change (FC) between the naïve (DMSO treated) and the TMZ treated cells was calculated. A probeset with FC > 1 for both the U251 and U87, max(expression value naïve, TMZ treated) > 6, and matching signs were selected. Probesets mapping to multiple genes or un-annotated was discarded. The final gene-level list was derived by slecting a single probeset for each gene. This resulted in 129 genes where 84 mapped to the Affymetrix U133A chip.

The TCGA GBM Affymetrix U133A data were processed data and normalised as previously described^13,14^. Overall Survival (OS) for the TCGA GBM samples was retrieved from the publication by Liu *et al*.^36^. A log rank p-value was calculated and median cut was applied in all survival analysis. TCGA RNAseq data were downloaded from https://gdc.cancer.gov/about-data/publications/pancanatlas and log2 transformed.

The TCGA Level 1 methylation data were downloaded from: http://tcga-data.nci.nih.gov/tcga/dataAccessMatrix.htm. The data including normalization via internal controls were preprocessed followed by background subtraction through the methylumi R package from Bioconductor^37^. The calculated beta values were then extracted from the MethyLumiSet object following preprocessing. Average beta-value across selected probes for XAF1 and MGMT was correlated with RNAseq gene expression value. The selection of probes was based on their location (TSS1500, TSS200, 5UTR 1stExon) and their correlation to each other.

Gene set enrichment analysis was performed using GSEA^38,39^ using the HALLMARK^38–40^ genesets. Validation of the TMZ gene signature was done based on the approach by Berglund *et al*.^33^.

Bioinformatics analysis and figures were done using MATLAB 9.3 and Statistics and Machine Learning Toolbox 11.2, The MathWorks, Inc., Natick, Massachusetts, United States. Survival analysis was done in MatSurv (https://github.com/aebergl/MatSurv) and PCA was done using Evince (Prediktera AB, Umeå, Sweden).

### Statistics

Student’s t-test was used for all the studies unless indicated. p < 0.05 was considered a significant difference. *Means p < 0.05.

## Supplementary information

Supplementary Information

Dataset1

## Data Availability

The data that support the findings of this study are available from the corresponding authors upon reasonable request.
