# Supplementary material for: Paradoxical epigenetic regulation of XAF1 mediates plasticity towards adaptive resistance evolution in MGMT-methylated glioblastoma
Source: Sci Rep. 2019 Oct 1;9:14072. doi: 10.1038/s41598-019-50489-2 (PMC6773736; doi:10.1038/s41598-019-50489-2)
Supplement: Supplementary file 1 — Supplementary Information [file 41598_2019_50489_MOESM1_ESM.docx]

**Paradoxical epigenetic regulation of XAF1 mediates plasticity towards adaptive resistance evolution in MGMT-methylated glioblastoma**

**Qiong Wu^1^, Anders E. Berglund^2^, Dapeng Wang^1^, Robert J. MacAulay^3^, James J. Mulé^4^, and Arnold B. Etame^1*^**

Departments of ^1^Neuro-Oncology, ^2^Biostatistics and Bioinformatics,^3^Anatomic Pathology, and ^4^Immunology, H. Lee Moffitt Cancer Center and Research Institute, 12902 Magnolia Drive, Tampa, FL 33612 USA.

*Corresponding author:

|  |
| --- |

Arnold B. Etame MD PhD

Department of Neuro-Oncology

Moffitt Cancer Center and Research Institute

12902 Magnolia Drive, Tampa, FL 33612

Tel: +1-813-745-3871

E-mail: [arnold.etame@moffitt.org](mailto:arnold.etame@moffitt.org)

**Supplementary information:**

**Supplementary Methods and Materials**

**Reagents**

Temozolomide (TMZ) was obtained from Sigma-Aldrich. G418 sulfate was obtained from Life Technologies (USA). Control siRNA and XAF1 siRNA was obtained from Sigma-Aldrich (USA). Rabbit anti-Human XAF1, Goat anti-Rabbit IgG-HRP, Goat anti-mouse IgG-HRP and anti-β-actin IgG-HRP were obtained from Santa Cruz Biotech (USA); Rabbit anti-Human MGMT were obtained from Novus (USA). iQ SYBR Green supermix was obtained from Bio-Rad (USA).

**siRNA knock down**

U251 and T98G cells were transfected with XAF1 small interfering RNA (siRNA; 80 nM, Sigma-Aldrich) or control siRNA (80 nM) using siRNA transfection reagent (Santa Cruz, USA). Briefly, one day prior to transfection, the cells were seeded in 6-well plate (2 × 10^5^) with 10% FBS DMEM without antibiotics. siRNAs were prepared according to the manufacturer’s instructions and added to the cells. The medium was replaced with DMEM containing 10% FBS 6 hours after transfection, and the cells were then exposed to TMZ after transfection prior to analysis.

**Gene silencing by CRISPR/Cas9 system**

CRISPR/Cas9 vectors lentiCRISPR-v2-puro was obtained from Addgene. sgRNA targeting human XAF1 and sgRNA control were cloned into lentiCRISPR-v2-puro. XAF1 targeting forward primer: CACCGAGCATGCAGAAGTCCTCGC, reverse primer: AAACGCGAGGACTT CTGCATGCTC; control sgRNA cloning forward primer: CACCGCACTCACATCGCTACATCA, reverse primer: AAACTGATGTAGCGATGTGAGTGC. Lentivirus was packed by 293T cells through 2^nd^ generation lentivirus packaging system. U251 and T98G cells were next infected with Lenti-sgXAF1-puro or Lenti-sgCTL-puro followed by extensive selection with 1μg/ml puromycin (InvivoGen). Gene silencing efficiency was examined by western blot. To confirm CRISPR editing efficiency, we harvested genomic DNA from U251 and T98G cells, and cloned target region of XAF1 into T-vector. 10 clones were picked from each cell line for Sanger Sequencing.

**Western-blot analysis**

50-100 μg of heat-denatured proteins were loaded on 4–15% precast polyacrylamide gel (Bio-Rad, USA). The proteins were then transferred to PVDF membranes (Bio-Rad, USA.), which were blocked with 5% non-fat milk solutions for 1 hour at room temperature. The target proteins were then detected by the primary antibody at 4 °C overnight, washed with 0.1% Tween-TBS and incubated with appropriate secondary antibody for 1 hours at room temperature. The membranes were then washed and the target proteins were detected with luminol reagent and X-ray film (Santa Cruz).

**
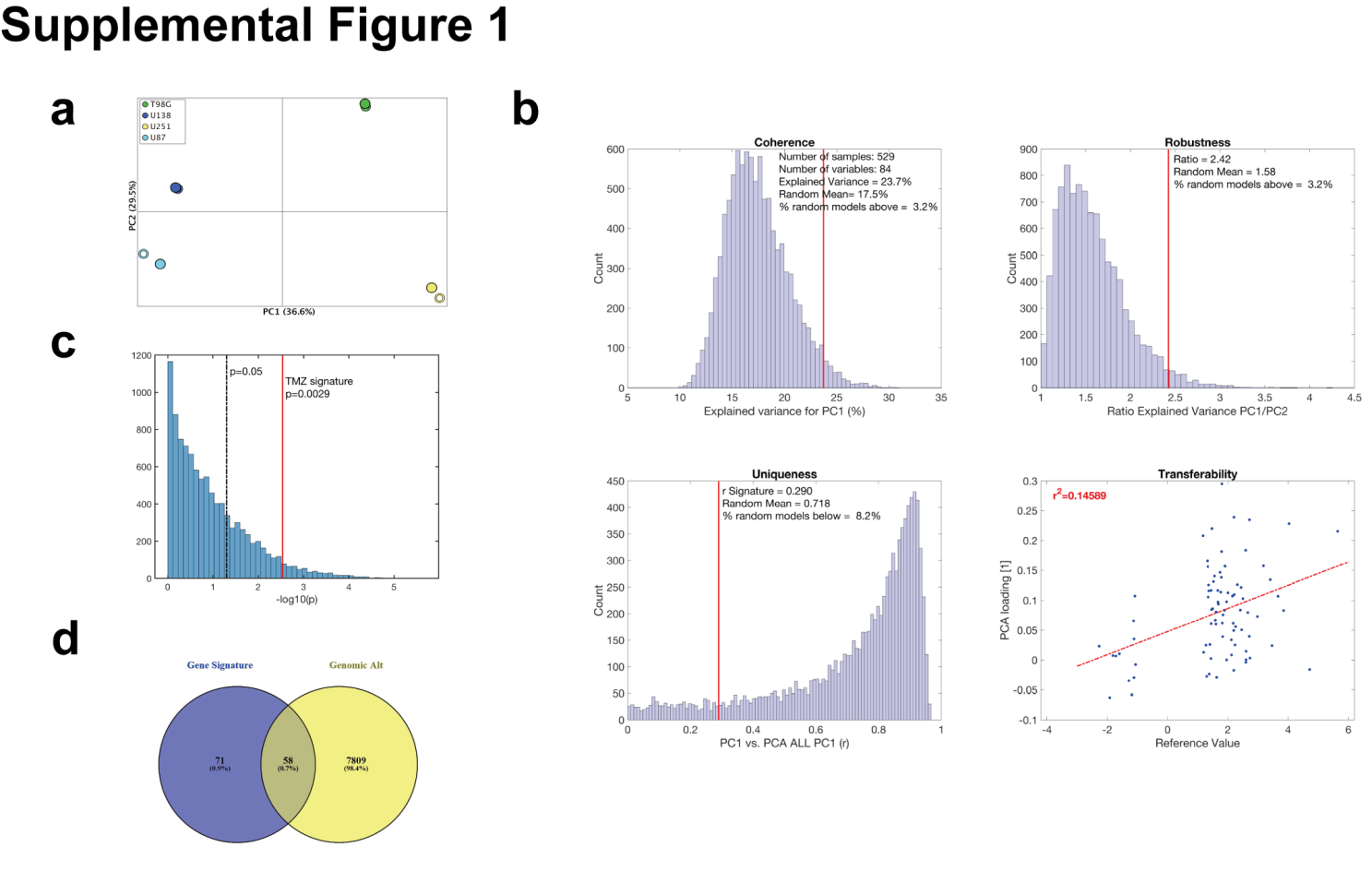
Supplementary Figures and legends:**

**Supplementary Figure 1. PCA data Gene signature analysis in TCGA GBM data.** (a) PCA plot illustrates shows only small changes due to the treatment; most of the differences are attributable to the individual cell lines. (b) Validation of PCA signature in the TCGA GBM. Top left panel shows that the first principal component (PC1) used to summarize the expression of all the genes, explains more than most randomly generated gene signatures. Tha ratio of PC1/PC2 is also > 2 indicating that the signature is robust, top right panel. The lower left panel shows that the TMZ signature is different from the general direction in the TCGA GBM dataset, militating against a potential batch effect. Finally, in the lower right panel we show that the TMZ signature describes similar biology as the true TMZ gene signature. (c) Our signature has a lower log rank p-value than most random models. (d) Venny diagram of cross analysis our gene signatures with genomic alternation caused by long time TMZ treatment.


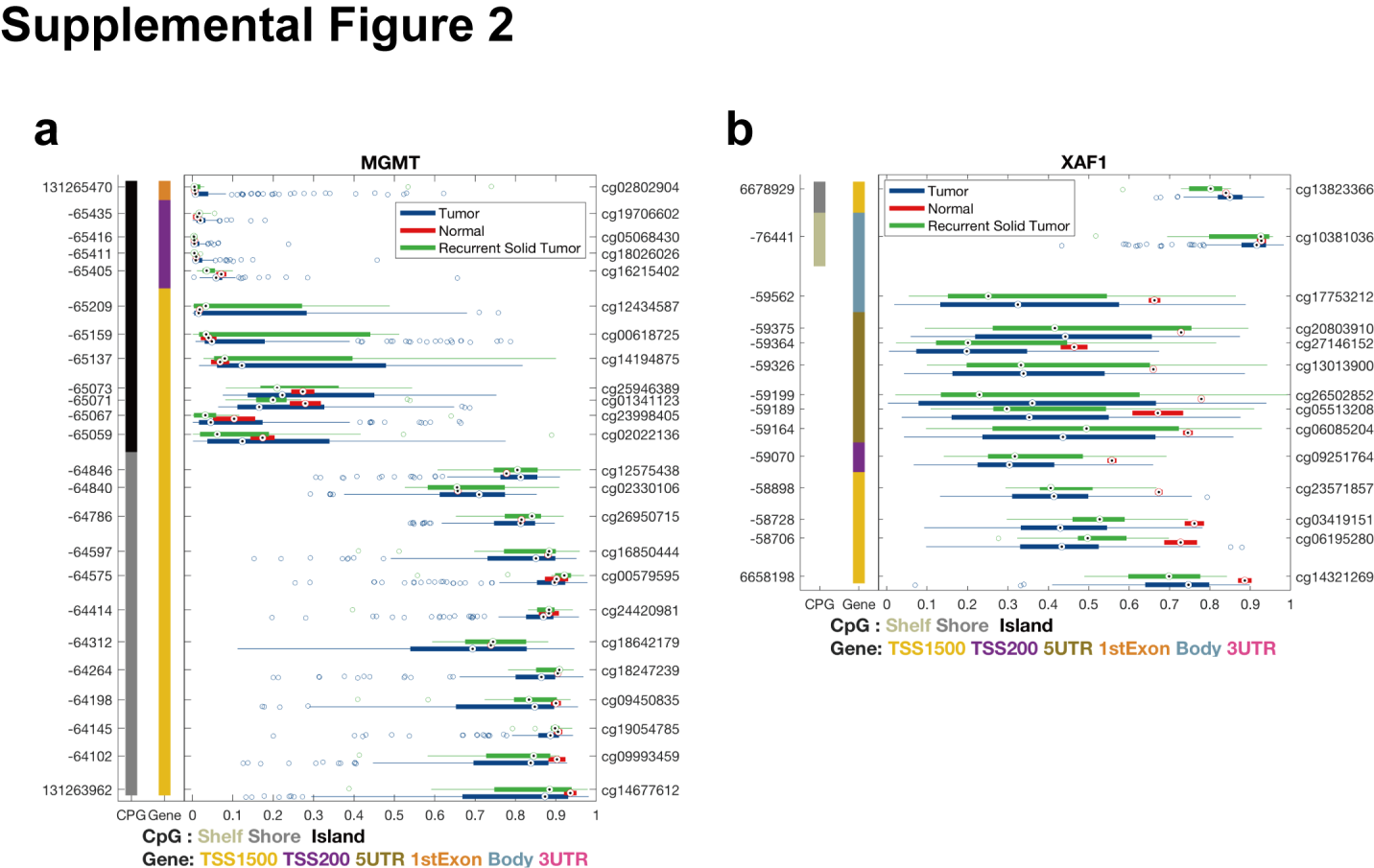


**Supplementary Figure 2. MGMT and XAF1 methylation analysis in TCGA GBM dataset.** The methylation for pattern for 24 selected probes (TSS1500, TSS200, 1^St^ Exon region) for MGMT are shown in (a) as boxplots for the the TCGA GBM dataset. The X-axis show the level of methylation ranging from zero to one. The y-axis shows the probe Id position (right axis) and genomic position (left axis) for all the probes. The colored columns indicate CpG island and gene body type as described in the figure. XAF1 methylation pattern are shown in (b) using a same type of figure.

**
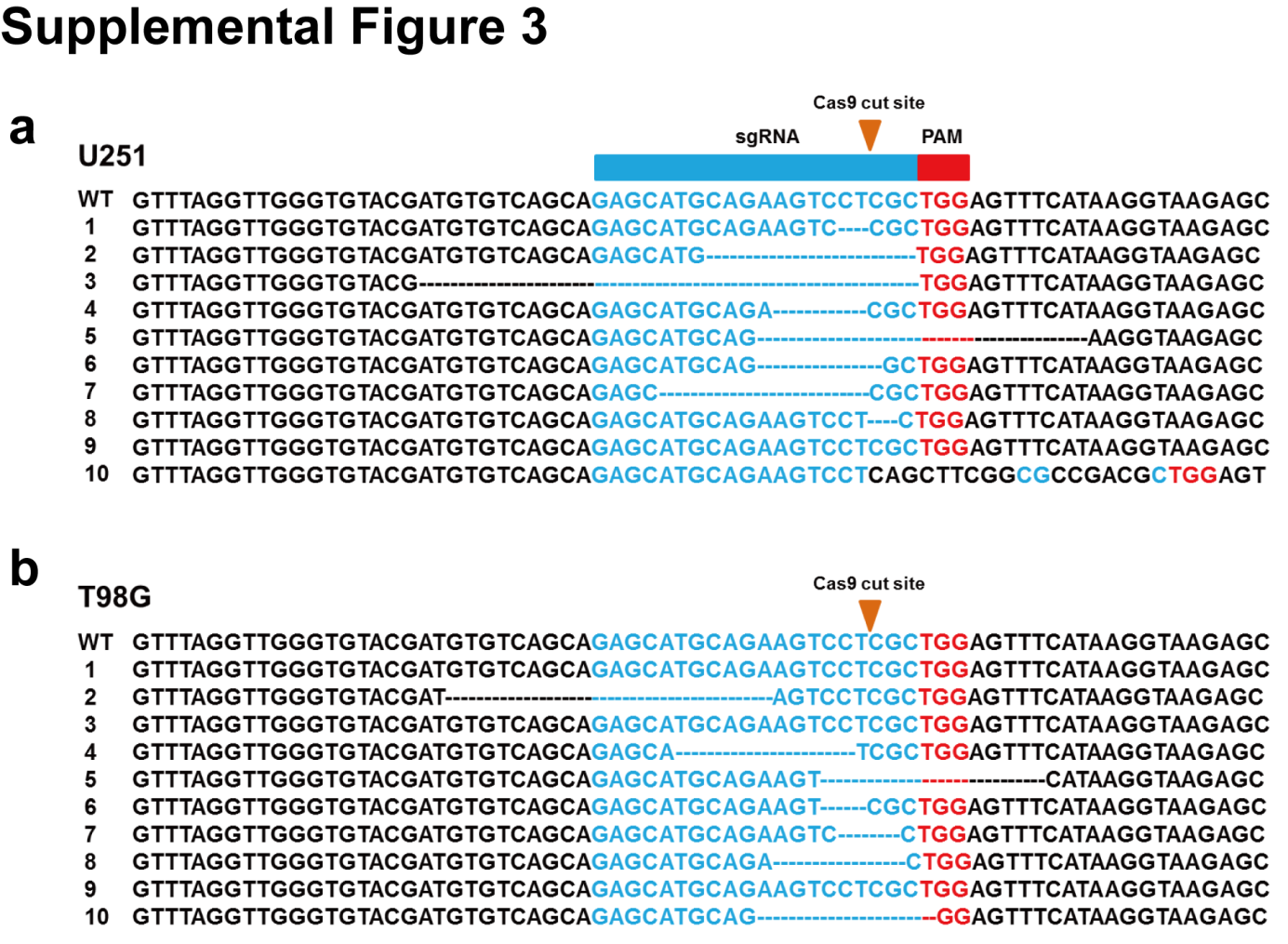
**

**Supplementary Figure 3. Gene editing at XAF1 locus by CRISPR/Cas9 system in U251 and T98G cells.** (a, b) Alignment of wild-type (WT) and XAF1 knockdown (by CRISPR-sgXAF1) cell sequences at XAF1 gene target region. Sequencing from each cell line indicated 10 clonal from T-vector (No.1-10).

**
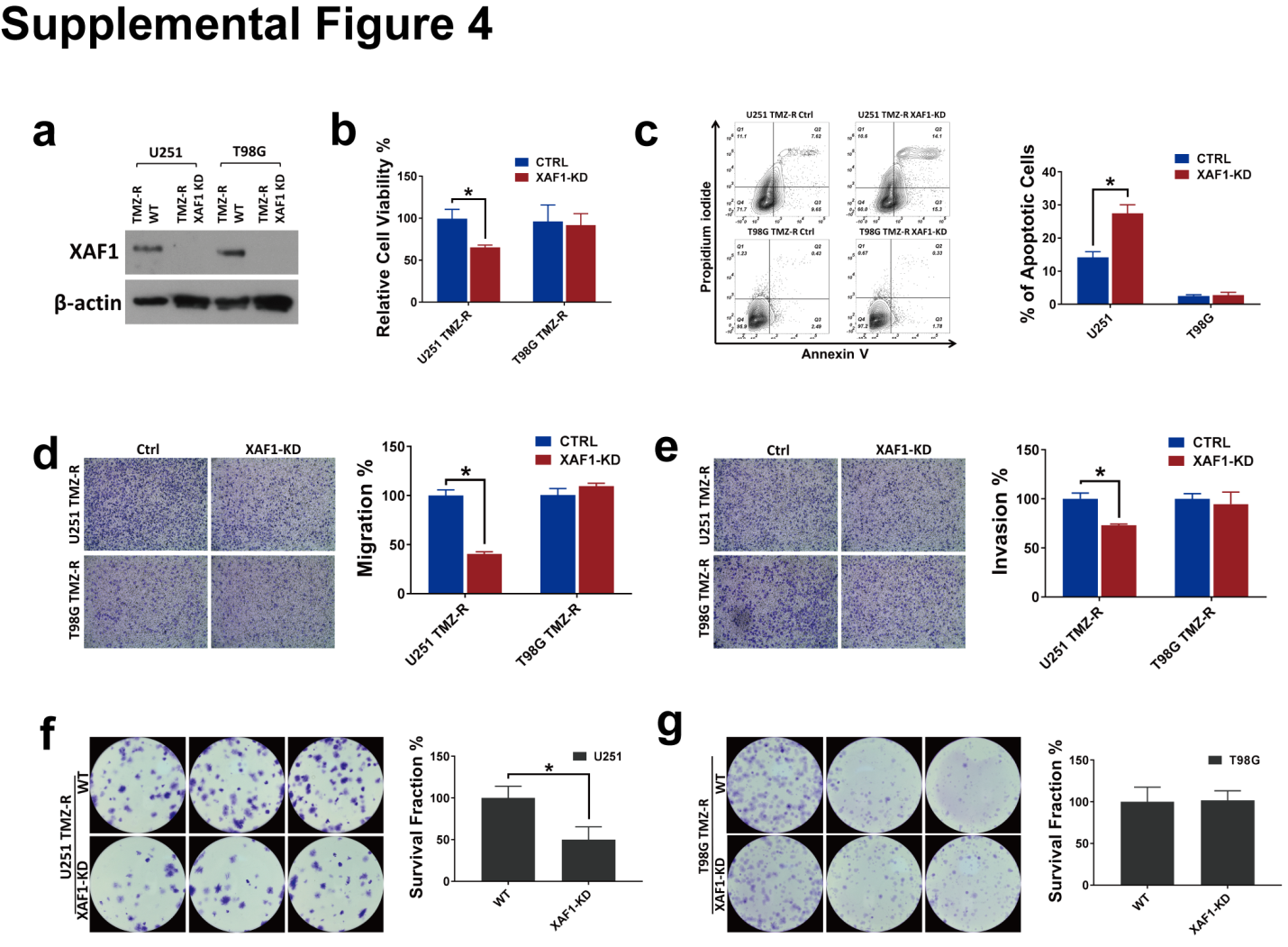
**

**Supplementary Figure 4. Loss function of XAF1 in resistant GBM cells leads to biological behavior changes.** (a) Western blot of XAF1 silencing (XAF1-KD) in U251 and T98G TMZ resistance cells. (b) 1 × 10^3^ U251-Resistance, T98G-Resistance control and XAF1 silenced cells (XAF1-KD) were seeded in 96 well plates. Cells were then treated with TMZ (50μM) for 5 days and cell viability was measured by the XTT Assay. The relative viability is shown. With significance, p=0.01. (c) Cells were seeded in 12 well plates overnight, 24 hours later treated with 50 μM of TMZ for 5 more days. Apoptosis was measured and quantified by Annexin V/PI staining through flow cytometry. With significance, p=0.007. (d) Trans-well migration assay of U251-R, T98G-R wild type and XAF1 silenced cells (XAF1-KD). With significance, p=0.002. (e) Trans-well invasion assay of U251-R, T98G-R control and XAF1 silenced cells (XAF1-KD). With significance, p=0.01. (f, g) The colony forming ability of U251, T98G control was compared with XAF1 silenced cells (XAF1-KD) in presence of 50μM TMZ. With significance, for U251, p=0.038. All experiments were performed in triplicate and error bar represent the mean ± SD; n = 3, with significance *p < 0.05 by Student’s t-test.

**Supplementary Full-length blots**

**
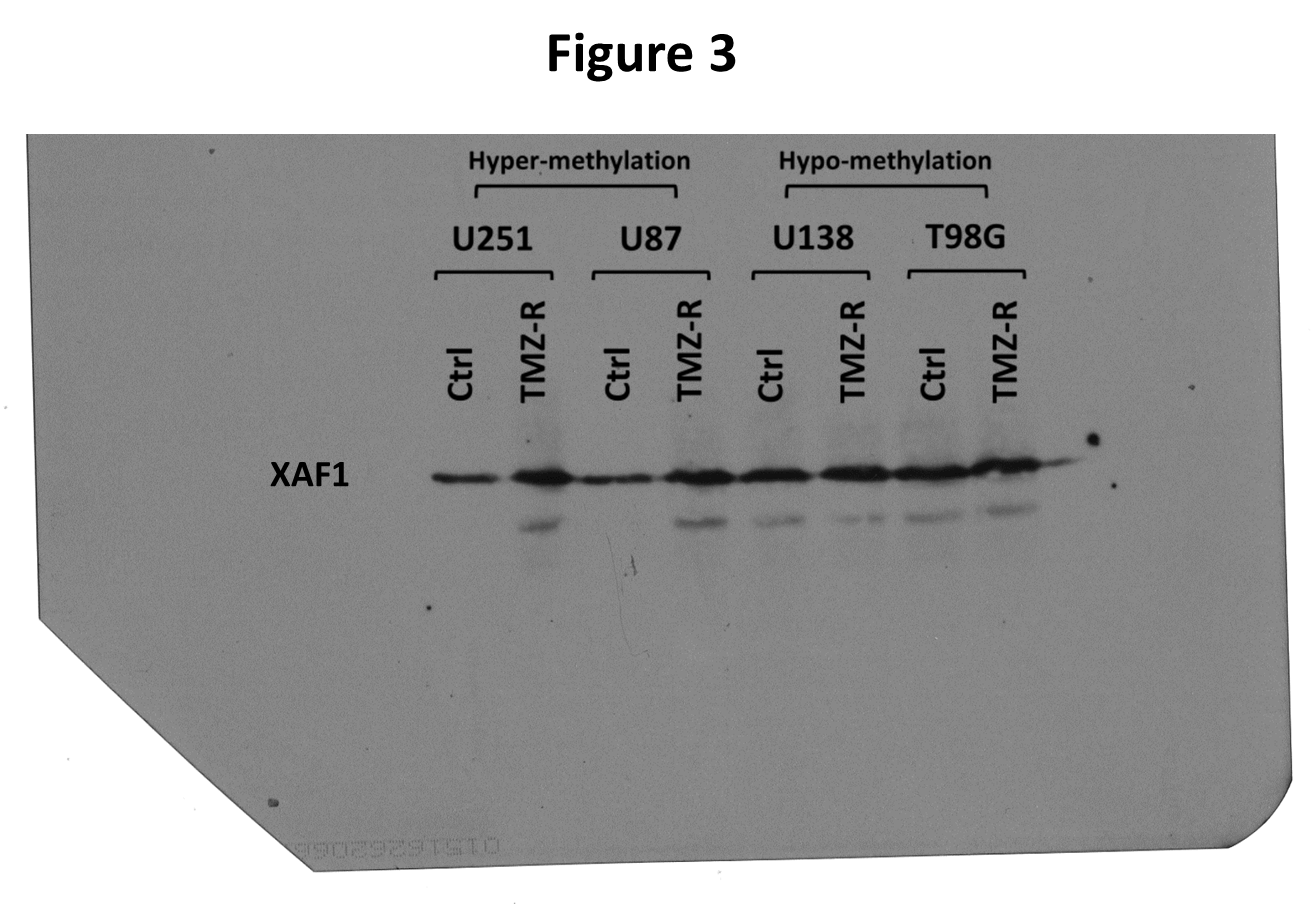
**

**
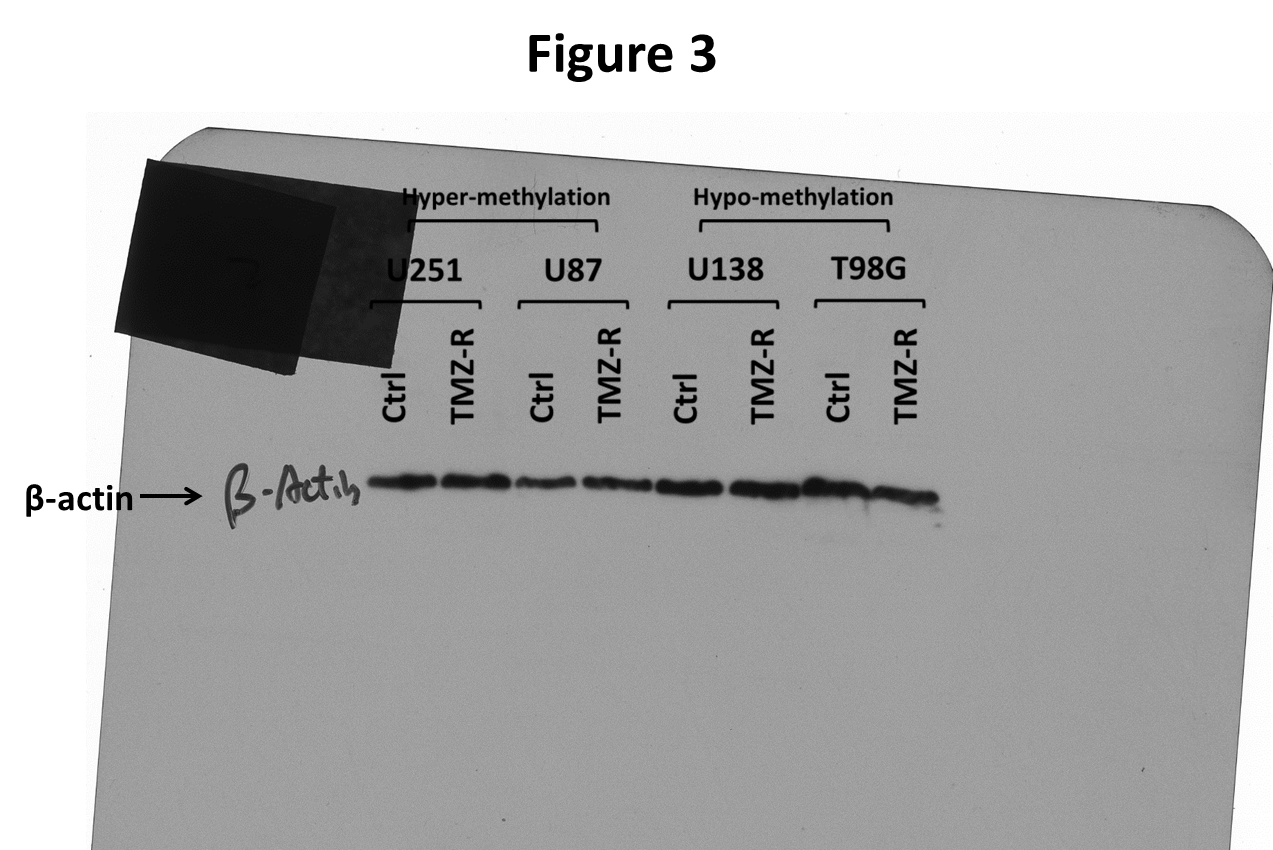
**

**
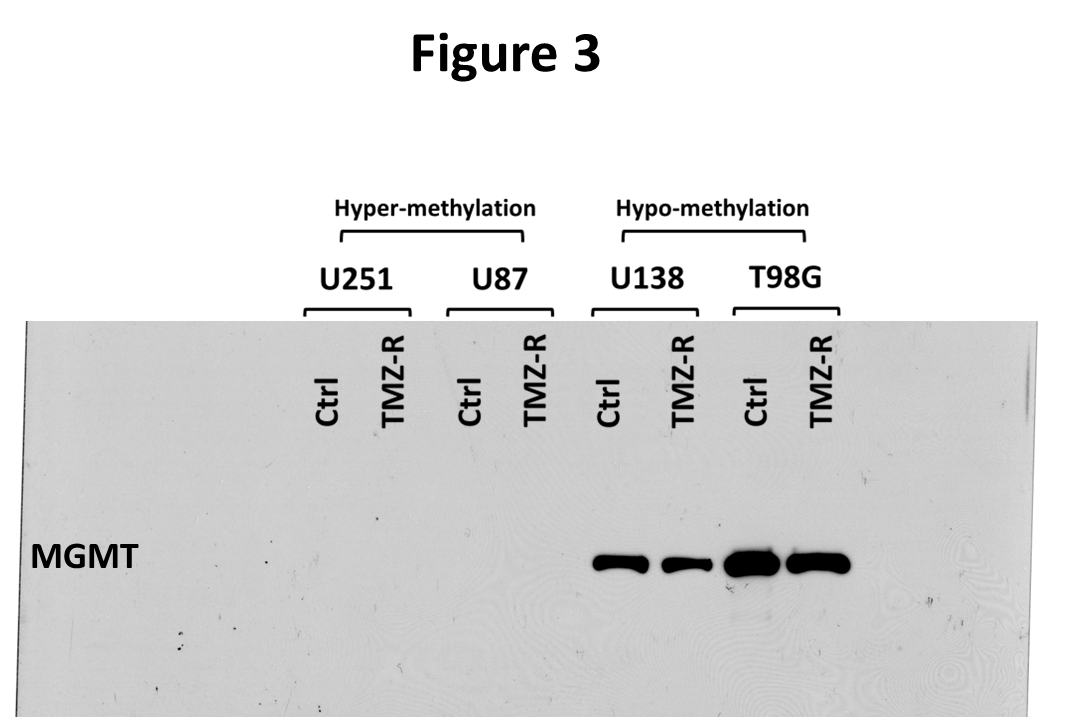
**

**
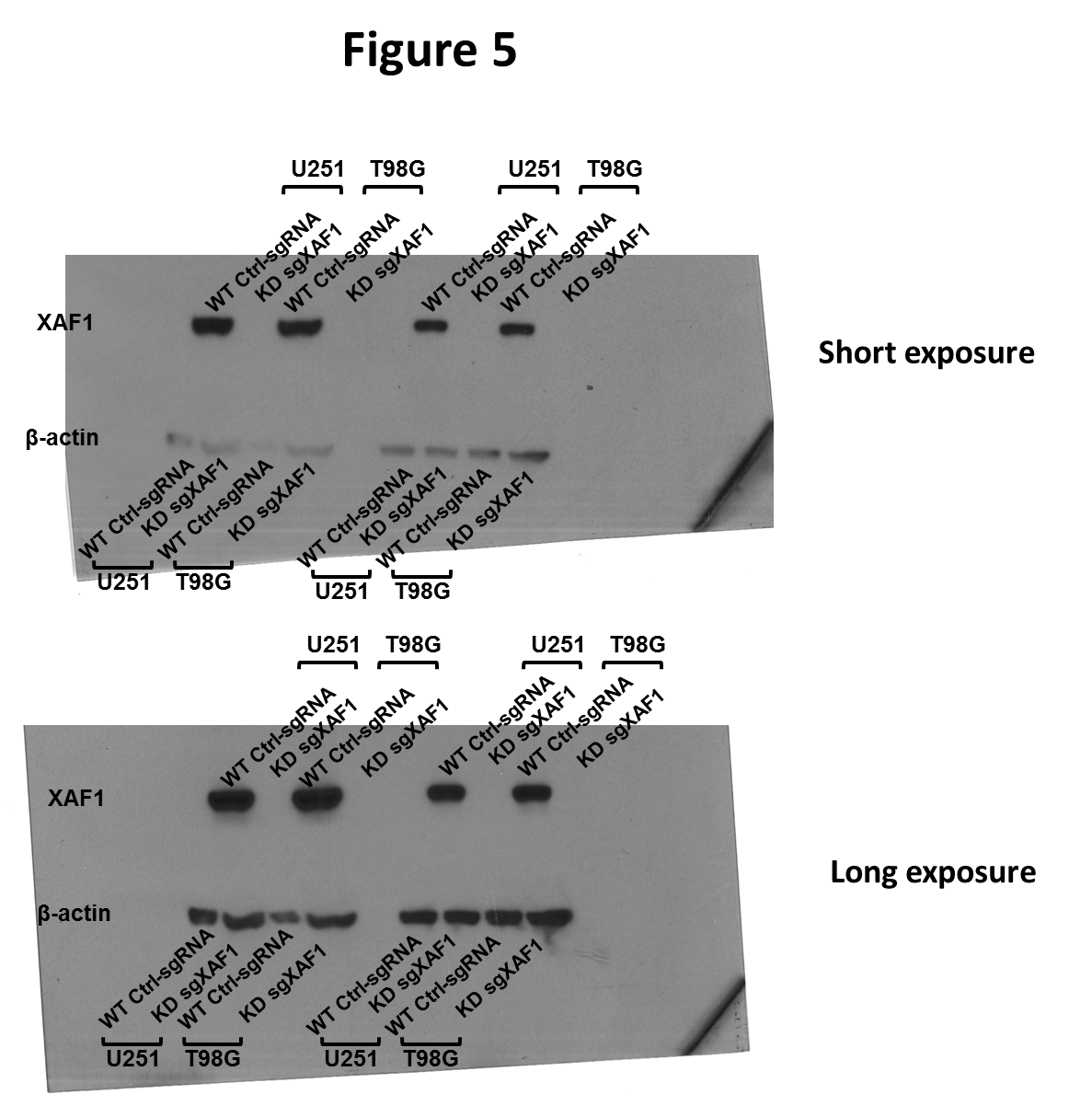
**

**
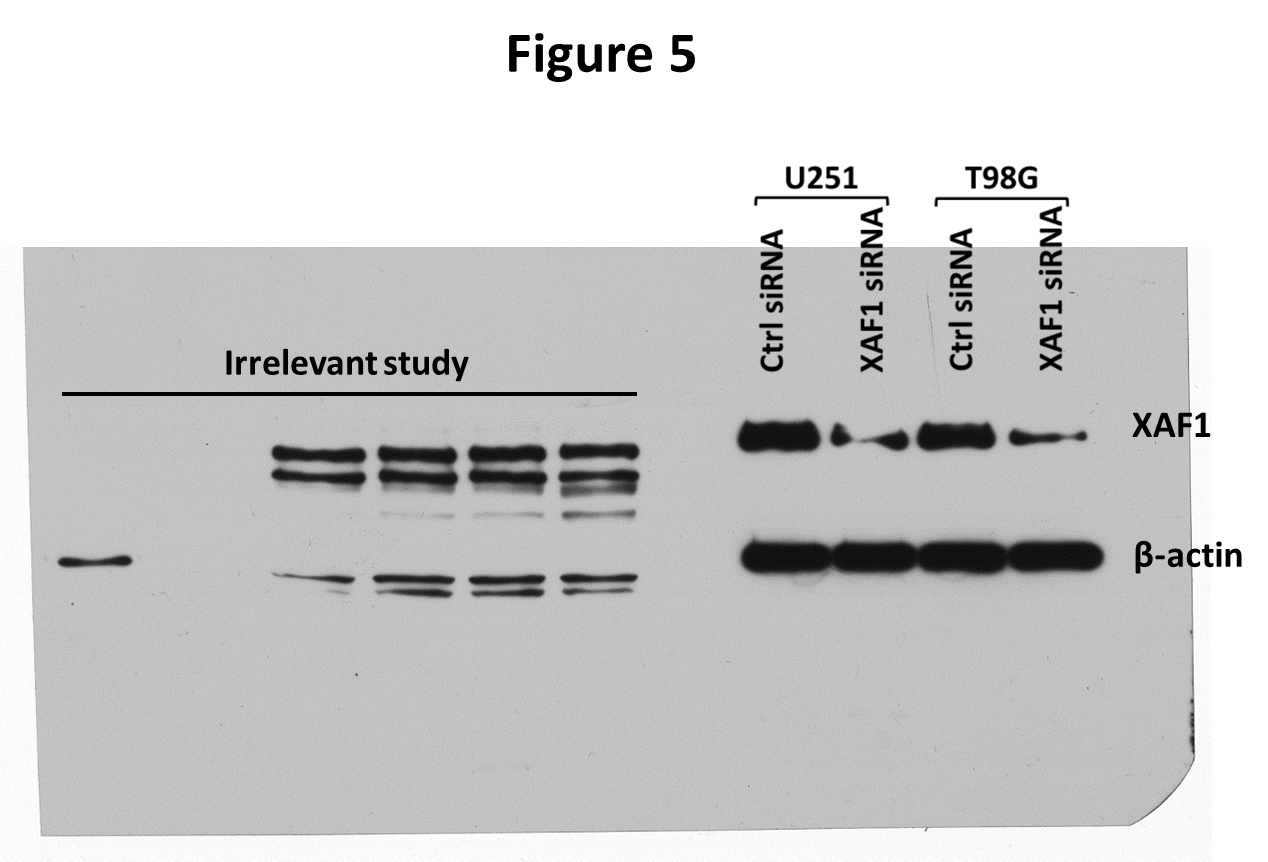
**

**
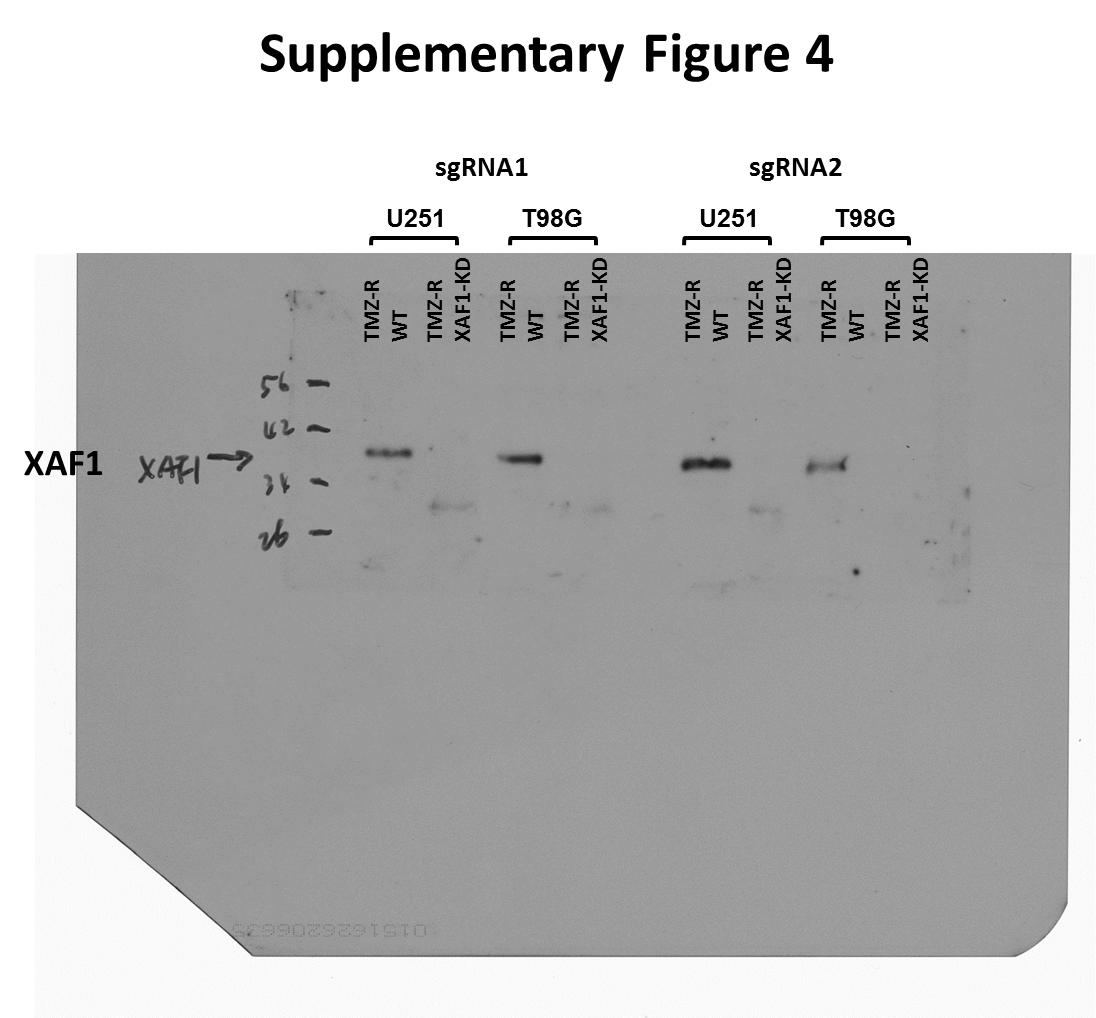
**

**
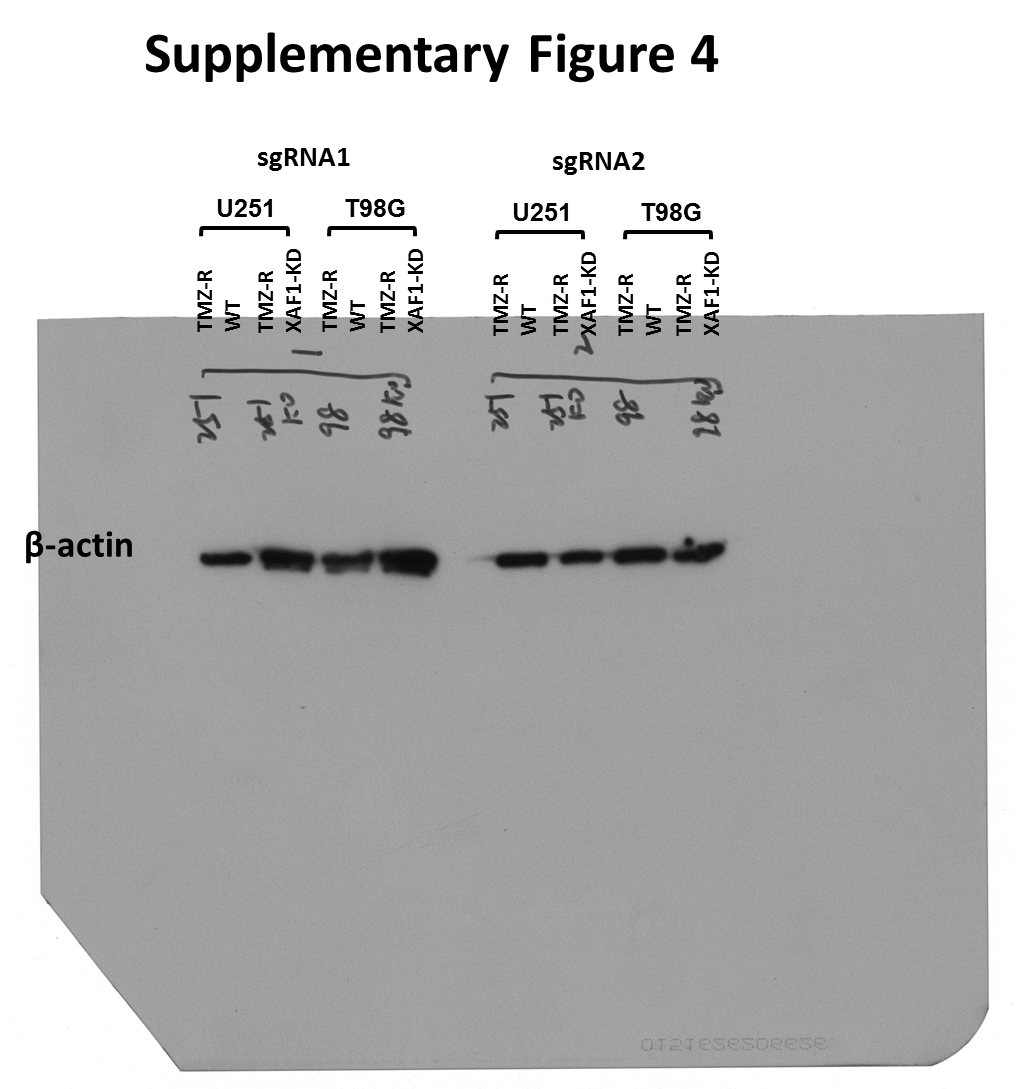
**
